# Progress in Ophthalmology

**Published:** 1900-01-20

**Authors:** 


### PROGRESS IN OPHTHALMOLOGY.

**Calomel in the Treatment of Conjunctivitis.**—Everyone, of course, has seen the successful results of calomel dusted on the eye in phlyctenular conjunctivitis, but Poukaloff[1] thinks that, in ophthalmia neonatorum, calomel will meet the same indications as nitrate of silver, and that it has not the same inconveniences as the silver salt. He advises the conjunctivæ to be carefully cleansed with a solution of boric acid, dried with tampons of cotton, and then the calomel dusted over the mucous membrane in a thin layer. The method is applicable among the poor, as it requires to be repeated only once a day, and in the vast majority of cases is followed by a prompt amelioration of the symptoms, the duration of many of the cases not exceeding seven days; even in those cases that were chronic and severe recovery has been noted in 15 days. The author's observations are based on 50 cases which he has treated.

**Epidemic of Follicular Conjunctivitis in a Liverpool School.**—Karl Grossman and Loewanthal,[2] at the Liverpool Medical Society, communicated a paper on an epidemic of follicular conjunctivitis. A case of advanced follicular conjunctivitis without marked subjective symptoms was discovered, and led to an examination of all the children of the school. It was then found that of the 700 children at the school 422 had follicular conjunctivitis. Mr. Shears, who also spoke, said that in his experience follicular conjunctivitis was an extremely common affection in young people, and was often accompanied by other signs of glandular activity such as post-nasal adenoids and granular conjunctivitis. Quoting Sydney Stevenson, who had examined the eyes of nearly 15,000 children in all ranks of life, and who had found enlarged follicles in over 80 per cent., "The majority of these children did not complain of their eyes in any way." While quite agreeing that there was no difficulty in distinguishing between the ordinary cases of follicular conjunctivitis and true granular lids, he thought that there were certain severe cases of follicular conjunctivitis which were very difficult, if not impossible, to differentiate from true granular lids, and that only time and careful observation could clear up the diagnosis.

**Correction of Ametropia in Treatment of Trachoma.**—Arnold Lawson,[3] in a paper read before the British Medical Association at Portsmouth bearing the above title, having quoted two cases of this disease which had resisted all the routine forms of treatment, and which eventually were found to have a high degree of mixed astigmatism, says that after correction of the ametropia and the wearing of glasses for six months they were almost if not perfectly well, that the patients were able to resume their employment and had no further trouble from the eyes; local applications were of course kept up during this time. In conclusion, he asks as to the second point, May one conceive that high ametropia may modify or in any way influence the special pathological processes that we associate with trachoma? Can it influence in any way the formation of the trachomatous granulations?

I think one may fairly infer something by comparing follicular conjunctivitis and trachoma. Not that the former is ever the cause of the latter, but that the follicle in the one and the granule in the other have much in common, and have a similar commencement. The one is a hypertrophied lymph follicle, and the other this, with some further modification. I need not dwell upon the connection between follicular inflammation and ametropia. The two are so frequently associated together, and it so often happens the correction of the one cures the other, that a distinct pathological connection cannot be doubted in a large number of cases, excluding, of course, that particular variety of follicular inflammation which occurs in epidemic form. Moreover, it is not hard to see why enlarged follicles and ametropia should go together. Follicular inflammation in connection with ametropia is encountered almost, if not entirely, in young subjects, most frequently under the age of puberty. Children are very apt to suffer from overgrowth of lymphoid tissue from slight chronic irritation. A slight but prolonged and constant congestion of the eyes frequently accompanies the overstrain induced by high ametropia, and this chronic increase in vascularity acts as an irritant, and induces the changes which we associate with follicular inflammation. And if ametropia may do so much in the causation of follicular inflammation, why should it not exercise some influence upon the growth and exuberance of the trachoma granule, which after all is but an exaggerated variety of adenoid hypertrophy? Such a question does not trespass on the domain of a primary specific origin for trachoma. A specific virus may initiate the growth, while secondary influences may promote or retard its progress.

[1] New York Med. Jour., Aug. 19, 1899. [2] Lancet, Nov. 11, 1899. [3] Brit. Med. Jour., Sept. 23, 1899.

